# Supplementary material for: Enterohaemorrhagic E. coli utilizes host- and microbiota-derived L-malate as a signaling molecule for intestinal colonization
Source: Nat Commun. 2023 Nov 9;14:7227. doi: 10.1038/s41467-023-43149-7 (PMC10636207; doi:10.1038/s41467-023-43149-7)
Supplement: Supplementary file 1 — Supplementary Information [file 41467_2023_43149_MOESM1_ESM.pdf]

Supplementary Information

Enterohaemorrhagic *E. coli* utilizes host- and microbiota-derived L-malate as a signaling molecule for intestinal colonization

Bin Liu [1,2,3], Lingyan Jiang [1,2,3], Yutao Liu [1,2,3], Hongmin Sun [1,2], Jun Yan [1,2], Chenbo Kang [1,2] & Bin Yang [1,2,*]

[1] TEDA Institute of Biological Sciences and Biotechnology, Nankai University, TEDA, Tianjin 300457, P. R. China

[2] The Key Laboratory of Molecular Microbiology and Technology, Ministry of Education, Tianjin 300071, P. R. China

[3] These authors contributed equally: Bin Liu, Lingyan Jiang, Yutao Liu.

[*] Correspondence and requests for materials should be addressed to B.Y. (email: yangbin@nankai.edu.cn

This Supplementary Information includes：

Supplementary Figures 1 to 7

Supplementary Figures

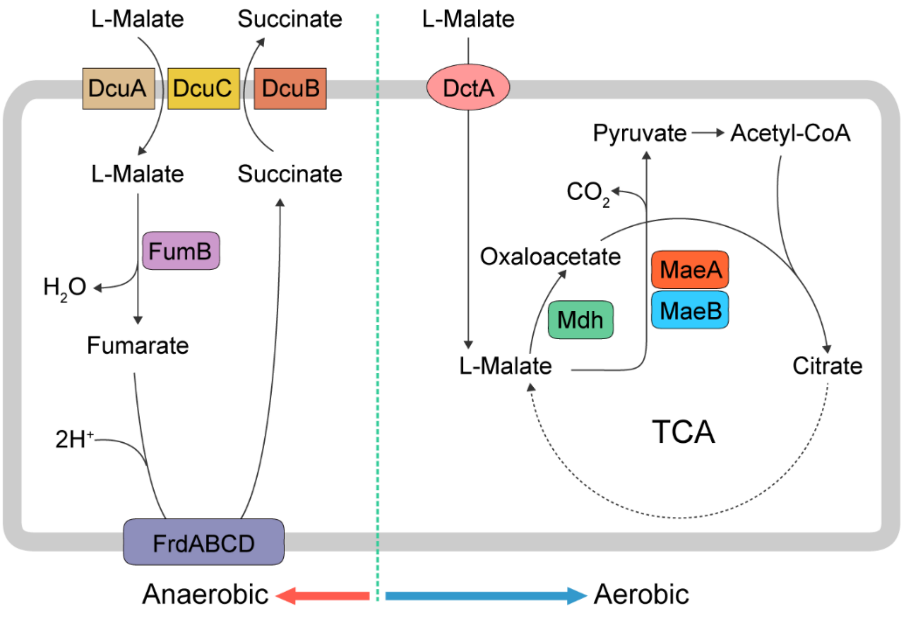

Supplementary Fig. 1 Different strategies for anaerobic and aerobic utilization of L-malate by EHEC O157:H7 using fumarate respiration and the TCA cycle.

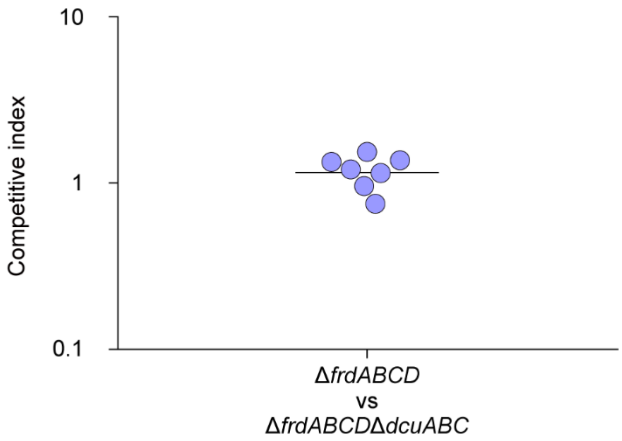

Supplementary Fig. 2 L-malate internalized through the DcuABC transporter is catabolized by fumarate respiration using FrdABCD. Competition index analysis between the Δ*frdABCD* and Δ*frdABCD*Δ*dcuABC* mutants. The horizontal line indicates the geometric mean; n = 7 infant rabbits were used. Source data are included in Source Data file.

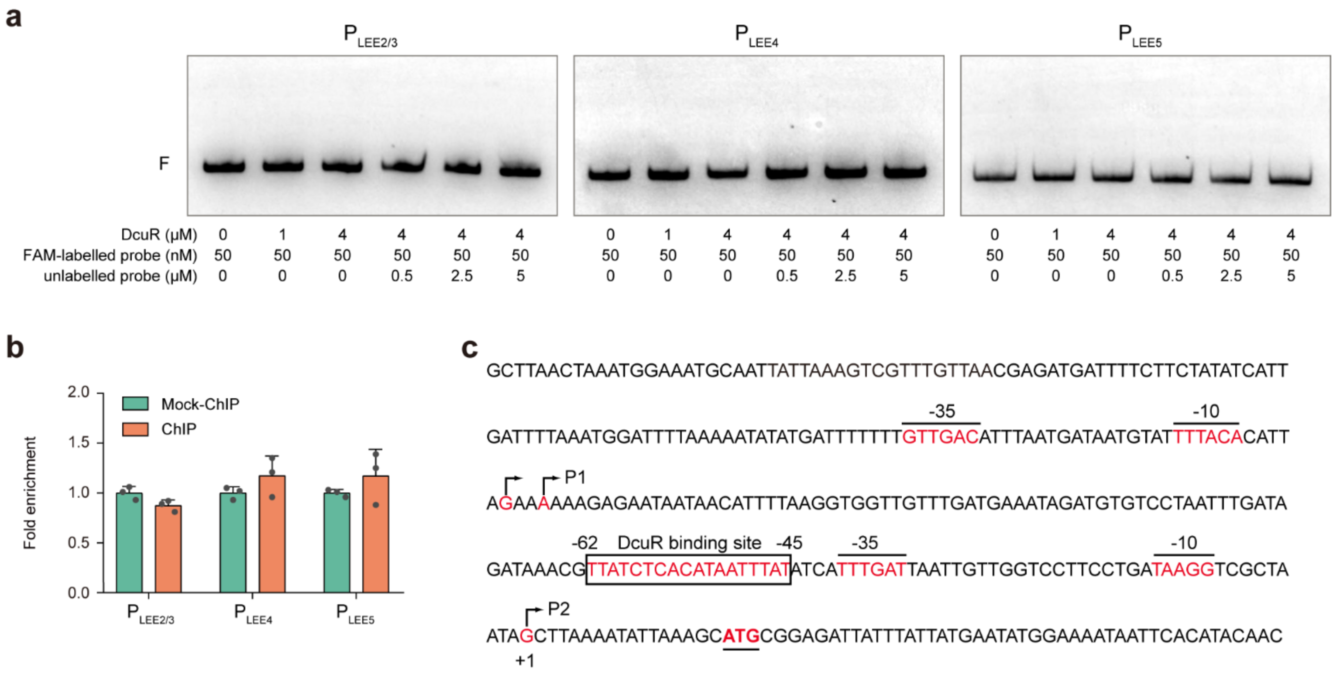

Supplementary Fig. 3 DcuR did not bind to the promoter regions of LEE2/3, LEE4 and LEE5 *in vitro* or *in vivo*. a Gel mobility shift and competition assays of DcuR with the promoter regions of LEE2/3, LEE4, and LEE5. Positions of the free (denoted with “F”) probes are shown on the left, and the concentrations of the probe and purified DcuR are indicated at the bottom of each lane. Images are representative of three independent experiments. **b** Fold enrichment of the promoter regions of LEE2/3, LEE4, and LEE5 in DcuR-ChIP samples, as measured via ChIP–qPCR. The data are presented as the mean ± SD of three independent biological replicates (n = 3). **c** DNA sequence of the LEE1 (*ler*) promoter region, including the -10 and -35 recognition sequences and transcriptional and translational start sites. The rectangle indicates the sequence protected by DcuR in the DNase I footprinting assay. Source data are included in Source Data file.

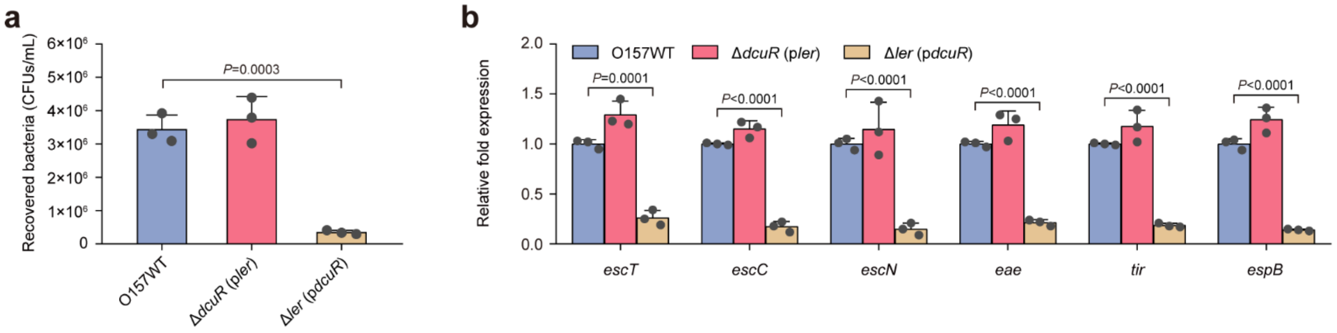

Supplementary Fig. 4 The virulence defects of the Δ*dcuR* mutant could be restored by complementation with *trc* promoter-controlled *ler*. a Adherence of O157 WT, Δ*dcuR*(p*ler*) and Δ*ler*(p*dcuR*) to HeLa cells. **b** qRT–PCR to determine LEE gene expression changes in O157 WT, Δ*dcuR*(p*ler*) and Δ*ler*(p*dcuR*). O157 WT, EHEC O157:H7 wild-type strain; Δ*dcuR*(p*ler*), *dcuR* mutant complemented with *trc* promoter-controlled *ler*; Δ*ler*(p*dcuR*), *ler* mutant complemented with *trc* promoter-controlled *dcuR*. The data are presented as the mean ± SD of three independent biological replicates (n = 3). Statistical significance was assessed via one-way ANOVA followed by Dunnett's *post hoc* test (**a**) or two-way ANOVA followed by Dunnett's *post hoc* test (**b**). Source data are included in Source Data file.

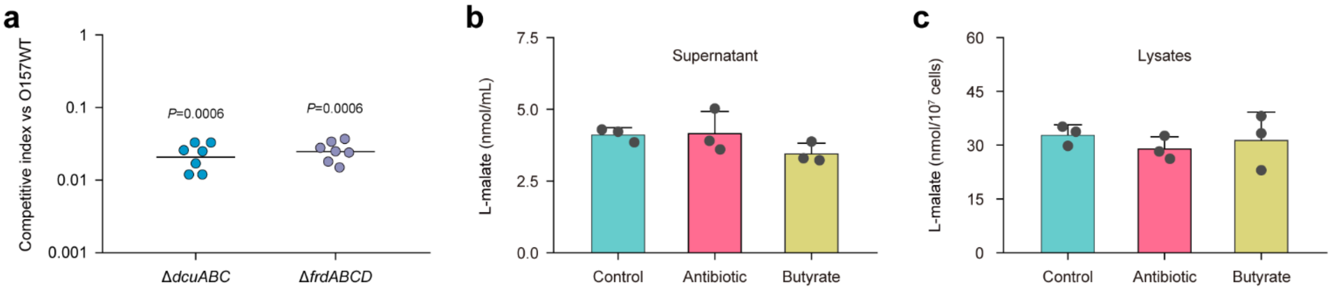

Supplementary Fig. 5 L-malate in the intestine of infant rabbits originates from both the host and the microbiota. a Competition index analysis between O157 WT and the Δ*dcuABC* mutant or Δ*frdABCD* mutant using germ-free infant rabbits fed an L-malate-free diet. The horizontal lines indicate the geometric mean for each group; n = 7 infant rabbits per group were used. Statistical significance was assessed via the two-sided Mann–Whitney rank-sum test. **b-c** Quantification of L-malate concentrations in the supernatants (**b**) and lysates (**c**) of HeLa cells in DMEM supplemented with a cocktail of four antibiotics (ampicillin, neomycin, metronidazole, and vancomycin; 100 μg/mL each) or 10 mM butyrate. The data are presented as the mean ± SD of three independent biological replicates (n = 3). Source data are included in Source Data file.

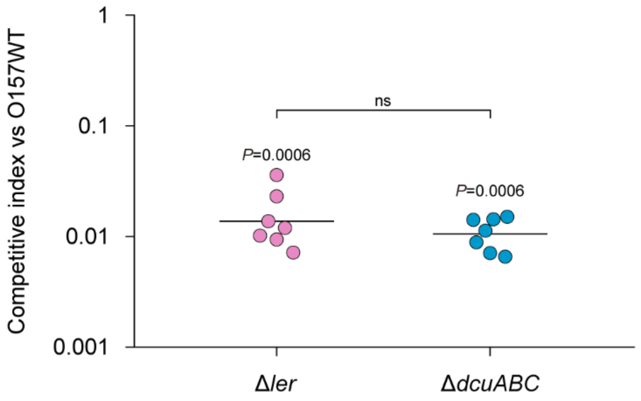

Supplementary Fig. 6 L-malate acts as a signal molecule and as a nutrient source and is equally critical for successful colonization of EHEC O157:H7 in the high-L-malate large intestine. Competition index analysis between O157 WT and the Δ*ler* mutant or Δ*dcuABC* mutant. The horizontal lines indicate the geometric mean for each group; n = 7 infant rabbits per group were used. Statistical significance was assessed via the two-sided Mann–Whitney rank-sum test. Source data are included in Source Data file.

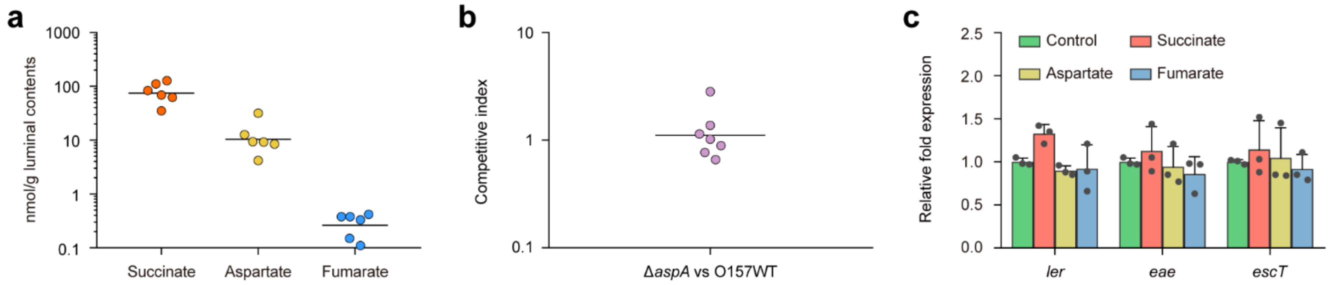

Supplementary Fig. 7 EHEC O157:H7 mainly utilizes L-malate rather than other C4-dicarboxylates (aspartate, fumarate and succinate) as an essential nutrient and signaling molecule for intestinal colonization. a Quantification of the concentrations of succinate, aspartate and fumarate in the colonic contents obtained from infant rabbits. **b** Competition index analysis between O157 WT and the Δ*aspA* mutant. For (**a**) and (**b**), the horizontal lines represent the geometric means; n = 7 infant rabbits per group were used. **c** qRT–PCR to determine LEE gene expression changes in O157 WT grown in DMEM supplemented with 750 μM succinate, 100 μM L-aspartate or 2.5 μM fumarate (corresponding to the concentrations in the colon of infant rabbits). The data are presented as the mean ± SD of three independent biological replicates (n = 3). Source data are included in Source Data file.
