# Supplementary material for: Enterohaemorrhagic E. coli utilizes host- and microbiota-derived L-malate as a signaling molecule for intestinal colonization
Source: Nat Commun. 2023 Nov 9;14:7227. doi: 10.1038/s41467-023-43149-7 (PMC10636207; doi:10.1038/s41467-023-43149-7)
Supplement: Supplementary file 6 — Reporting Summary [file 41467_2023_43149_MOESM6_ESM.pdf]

nature portfolio

Corresponding author(s): Bin Yang
Last updated by author(s): Oct 19, 2023

Reporting Summary

Nature Portfolio wishes to improve the reproducibility of the work that we publish. This form provides structure for consistency and transparency in reporting. For further information on Nature Portfolio policies, see our Editorial Policies and the Editorial Policy Checklist.

Statistics

For all statistical analyses, confirm that the following items are present in the figure legend, table legend, main text, or Methods section.

n/a	Confirmed	
☐	☒	The exact sample size (*n*) for each experimental group/condition, given as a discrete number and unit of measurement
☐	☒	A statement on whether measurements were taken from distinct samples or whether the same sample was measured repeatedly
☐	☒	The statistical test(s) used AND whether they are one- or two-sided<br>*Only common tests should be described solely by name; describe more complex techniques in the Methods section.*
☒	☐	A description of all covariates tested
☐	☒	A description of any assumptions or corrections, such as tests of normality and adjustment for multiple comparisons
☐	☒	A full description of the statistical parameters including central tendency (e.g. means) or other basic estimates (e.g. regression coefficient) AND variation (e.g. standard deviation) or associated estimates of uncertainty (e.g. confidence intervals)
☐	☒	For null hypothesis testing, the test statistic (e.g. *F*, *t*, *r*) with confidence intervals, effect sizes, degrees of freedom and *P* value noted<br>*Give P values as exact values whenever suitable.*
☒	☐	For Bayesian analysis, information on the choice of priors and Markov chain Monte Carlo settings
☒	☐	For hierarchical and complex designs, identification of the appropriate level for tests and full reporting of outcomes
☒	☐	Estimates of effect sizes (e.g. Cohen's *d*, Pearson's *r*), indicating how they were calculated

Our web collection on statistics for biologists contains articles on many of the points above.

Software and code

Policy information about availability of computer code

Data collection	Amersham Imager 600, Zeiss LSM800 confocal microscope, ABI 3730 Genetic Analyzer, QuantStudio™ 5 Real-Time PCR System
Data analysis	ImageJ, GraphPad Prism 8, Peak Scanner v1.0

For manuscripts utilizing custom algorithms or software that are central to the research but not yet described in published literature, software must be made available to editors and reviewers. We strongly encourage code deposition in a community repository (e.g. GitHub). See the Nature Portfolio guidelines for submitting code & software for further information.

Data

Policy information about availability of data

All manuscripts must include a data availability statement. This statement should provide the following information, where applicable:

- Accession codes, unique identifiers, or web links for publicly available datasets
- A description of any restrictions on data availability
- For clinical datasets or third party data, please ensure that the statement adheres to our policy

The metabolomic data generated in this study have been deposited in the MetaboLights database under accession code MTBLS8754 [www.ebi.ac.uk/metabolights/MTBLS8754]. All other data associated with this study are available in the main text and supplementary materials. Source data are provided with this paper.

Research involving human participants, their data, or biological material

Policy information about studies with human participants or human data. See also policy information about sex, gender (identity/presentation), and sexual orientation and race, ethnicity and racism.

Reporting on sex and gender	The study contains no human subjects
Reporting on race, ethnicity, or other socially relevant groupings	The study contains no human subjects
Population characteristics	The study contains no human subjects
Recruitment	The study contains no human subjects
Ethics oversight	The study contains no human subjects

Note that full information on the approval of the study protocol must also be provided in the manuscript.

Field-specific reporting

Please select the one below that is the best fit for your research. If you are not sure, read the appropriate sections before making your selection.

☒ Life sciences ☐ Behavioural & social sciences ☐ Ecological, evolutionary & environmental sciences

For a reference copy of the document with all sections, see nature.com/documents/nr-reporting-summary-flat.pdf

Life sciences study design

All studies must disclose on these points even when the disclosure is negative.

Sample size	All in vitro experiments were performed in duplicate and repeated at least three times (n ≥ 3). Animal experiments were conducted twice with at least 3 rabbit (n ≥ 3) in each group, and the combined data for the two experiments was used for statistical analysis.
Data exclusions	No data was excluded from analysis.
Replication	All the reported experiments were reproducible. Data reproducibility was confirmed by two or three independent experiments.
Randomization	Infant rabbits were randomized into the different experimental groups with same gender and similar weight. For in vitro experiments, samples were randomly allocated into different experimental groups.
Blinding	We were not blinded to the samples in this study when running all experiments. We were not blinded to the groups of infant rabbits in this study to prevent cross contamination.

Reporting for specific materials, systems and methods

We require information from authors about some types of materials, experimental systems and methods used in many studies. Here, indicate whether each material, system or method listed is relevant to your study. If you are not sure if a list item applies to your research, read the appropriate section before selecting a response.

Materials & experimental systems

n/a	Involved in the study	
☐	☒	Antibodies
☐	☒	Eukaryotic cell lines
☒	☐	Palaeontology and archaeology
☐	☒	Animals and other organisms
☒	☐	Clinical data
☒	☐	Dual use research of concern
☒	☐	Plants

Methods

n/a	Involved in the study	
☒	☐	ChIP-seq
☒	☐	Flow cytometry
☒	☐	MRI-based neuroimaging

Antibodies

Antibodies used	anti-FLAG antibody (1:2,500 dilution, Sigma: F1804), anti-DnaK antibody (1:5,000 dilution, Abcam: ab69617), goat anti-mouse IgG-

Antibodies used	HRP (1:5,000 dilution, Abcam: ab205719)
Validation	All antibodies were obtained commercially and were tested and validated by the respective company. All antibodies had validation statement provided on the website of the manufacturer.<br>anti-FLAG antibody: mouse monoclonal to FLAG-tag; suitable for WB, IP, ICC/IF, and IHC-Fr; reacts with FLAG-tag (DYKDDDDK).<br>anti-DnaK antibody: Mouse monoclonal to DnaK; suitable for WB; reacts with Escherichia coli.

Eukaryotic cell lines

Policy information about cell lines and Sex and Gender in Research

Cell line source(s)	HeLa and Caco-2 cells were purchased from the Shanghai Institute of Biochemistry and Cell Biology of the Chinese Academy of Sciences (Shanghai, China).
Authentication	No further authentication was performed.
Mycoplasma contamination	All cell lines were tested negative for mycoplasma contamination.
Commonly misidentified lines (See ICLAC register)	No commonly misidentified lines were used.

Animals and other research organisms

Policy information about studies involving animals; ARRIVE guidelines recommended for reporting animal research, and Sex and Gender in Research

Laboratory animals	Three-day-old female New Zealand White (NZW) rabbits were purchased from Beijing Huabukang Biological Technology Co., Ltd. (Beijing, China) and housed under standard laboratory conditions with a 12 h light/dark cycle, at a temperature of 24 ± 2 °C, and a relative humidity of 50 ± 5%. Infant rabbits were fed a L-malate-free diet (50 µL of sterilized water containing 10% lactose) or L-malate-rich diet (50 µL of sterilized water containing 10% lactose and 10 mM L-malate) at 6 h intervals.
Wild animals	The study did not involve wild animals.
Reporting on sex	Infant rabbits do not exhibit sex-specific responses to EHEC O157:H7 infection. However, female infant rabbits are slightly larger than male infant rabbits, which is convenient for experimental operation.
Field-collected samples	No field collected samples were used in the study.
Ethics oversight	The animal experimental protocols were approved by the Institutional Animal Care Committee at Nankai University, Tianjin, China (protocol number: SYDWLL-000168).

Note that full information on the approval of the study protocol must also be provided in the manuscript.
